# Teachers' Knowledge and Use of Evidenced-Based Practices for Students With Autism Spectrum Disorder in Saudi Arabia

**DOI:** 10.3389/fpsyg.2021.741409

**Published:** 2021-09-16

**Authors:** Abdulkarim Alhossein

**Affiliations:** Special Education Department, College of Education, King Saud University, Riyadh, Saudi Arabia

**Keywords:** autism spectrum disorder, evidenced-based practices, knowledge, use, teachers, Saudi Arabia

## Abstract

The evidenced-based practices (EBPs) movement in the field of special education began ~20 years ago. This study contributes to that literature. It investigates the teachers' knowledge and use of EBPs to teach students with autism spectrum disorder (ASD) in Saudi Arabia. The Teachers' Knowledge and Use of EBPs Survey was administered to 240 special education teachers. The participants generally reported a medium level of knowledge and use of EBPs for students with ASD. Female teachers' use of EBPs was greater than that of males, and teachers who attended more than five professional development programs reported greater use of EBPs than those that attended fewer programs. Knowledge and use of EBPs were related. Gender and professional development programs were predictors of teachers' use of EBPs for students with ASD. Teachers' knowledge of EBPs for students with ASD is a vital indicator of teachers' use of those practices, professional development programs can improve such knowledge and use, and teachers' use of EBPs for students with ASD could be improved by offering high-quality professional development programs.

## Introduction

Autism spectrum disorder (ASD) is defined as a neurodevelopmental disorder characterized by persistent deficits in social communication and interactions and displays of restricted repetitive behaviors, interests, or activities (American Psychiatric Association, 2013; Kakkar, 2019). These symptoms appear during early childhood and impair everyday functioning (American Psychiatric Association, 2013; Wadhera et al., 2021). ASD is one of the most common neurodevelopmental disorders that affect children (Leblanc et al., 2009), and there has been an increase in the number of individuals diagnosed with the condition (Leblanc et al., 2009; Stansberry-Brusnahan and Collet-Klingenberg, 2010; Odom et al., 2013; Wong et al., 2015). According to Maenner et al. (2020), ~1 in 54 children were identified to have ASD in the United States (US), a rate higher than the 1 in 150 children in 2000–2002. In addition, the estimates of the prevalence of ASD have approached 1% of the population in the US and that in all other countries, with similar results in child and adult samples (American Psychiatric Association, 2013). Many of these children with ADS are included in general education classrooms (Meindl et al., 2020).

The increased prevalence of ASD and the inclusion of students with ASD in general education classrooms (Meindl et al., 2020) have intensified the need for effective practices that can significantly affect the social and educational functioning of these students. Unfortunately, there is no cure for ASD (Wadhera and Kakkar, 2020) and no single intervention has been recommended universally (Stansberry-Brusnahan and Collet-Klingenberg, 2010). Thus, teaching students with ASD can be a difficult task, and fulfilling their needs is challenging for teachers (Hsiao and Sorensen Petersen, 2019) because many of the symptoms associated with ASD can adversely affect these students' learning abilities (Leblanc et al., 2009).

The research has emphasized using evidence-based practices (EBPs; Odom et al., 2013; Wong et al., 2015) to meet the significant needs of students with ASD in inclusive classrooms (Marder and deBettencourt, 2015). Research has indicated that a teacher's use of EBPs can significantly improve the outcomes of students with ASD and the lives of their families (Cook and Odom, 2013; Alexander et al., 2015; Marder and deBettencourt, 2015). In addition, legal requirements (e.g., the Individuals with Disabilities Education Improvement Act, 2004 and the Every Student Succeeds Act, 2015) require teachers to use EBPs to fulfill the needs of students with disabilities (Lauderdale-Littin and Brennan, 2018; Spooner et al., 2019). This effort has resulted in EBPs being a common term in special education (Cook et al., 2012). EPBs are defined as teaching programs, instructional strategies, or interventions supported by experimental research and professional wisdom that result in consistent positive outcomes in students (Burns and Ysseldyke, 2009; Marder and deBettencourt, 2015; Beam and Mueller, 2017). However, research has revealed that non-EBPs are being used by professionals in the field of special education despite an increase in the number of EBPs (Hess et al., 2008; Paynter and Keen, 2015). Notably, special education specialists have reported using ineffective instructional practices more often than some research-based practices (Cook and Odom, 2013).

Researchers have measured teachers' perceived frequency of using EBPs with students with disabilities in North America (Burns and Ysseldyke, 2009), Australia (Carter et al., 2011), and the Czech Republic (Carter et al., 2012) and reported the extensive use of EBPs such as direct instruction and applied behavior analysis. Notably, some non-EBPs (e.g., modality instruction) were commonly used, and applied behavior analysis had been used with similar frequency (Burns and Ysseldyke, 2009; Carter et al., 2011, 2012). Unfortunately, non-EBPs were frequently used in special schools and classrooms more than regular classrooms (Carter et al., 2012). Czech teachers used EBPs at a higher level than US and Australian teachers (Burns and Ysseldyke, 2009; Carter et al., 2011, 2012). However, these studies did not focus on a particular category of special education.

Other studies (Hess et al., 2008; Paynter and Keen, 2015; Paynter et al., 2017; Knight et al., 2019) have focused on ASD. In a survey study, less than 8% of teachers who taught children with ASD from preschool to twelfth grade were using EBPs, and several of the top-reported strategies used in the classroom were non-EBPs (Hess et al., 2008). Special education teachers of students with autism and/or intellectual disability reported daily use of a wide range of EBPs. Notably, some ineffective or harmful practices were used more frequently than EBPs were. Participants who received any type of training or resources related to the practice in the last year indicated significantly more use of that practice than those who did not receive any type of training or resources (Knight et al., 2019).

Professionals and paraprofessionals' knowledge and use of EBPs were explored in autism early intervention services in Australia. They had a higher level of knowledge and use of EBPs than of non-EBPs (Paynter and Keen, 2015; Paynter et al., 2017). However, professionals reported higher levels of knowledge (Paynter and Keen, 2015; Paynter et al., 2017) and use (Paynter and Keen, 2015) of EBPs than paraprofessionals did. There was a significant association between the knowledge and use of EBPs, with knowledge level significantly predicting the use of EBPs (Paynter and Keen, 2015; Paynter et al., 2017).

In Saudi Arabia, research indicated that teachers reported low use of peer-mediated and self-mediated interventions. Female teachers were more knowledgeable about EBPs than male teachers. Major, educational level, and years of teaching experience were not significantly related to their knowledge of EBPs. However, there was a strong positive relationship between knowledge and use of EBTPs (Alhossein, 2016).

Teachers require adequate knowledge of EBPs and the skills necessary to implement them with their students (Marder and deBettencourt, 2015). Researchers have suggested several rationales for using EBPs with students with ASD, for example, the increasing number of students with ASD in schools, risks associated with disappointing outcomes for students with ASD and their families, and the history of non-EBPs that have been offered to the public (Marder and deBettencourt, 2015). Almost two decades have passed since the beginning of the EBP movement in the field of special education. The literature review revealed several evaluations of teachers' knowledge and use of EBPs in developed countries (e.g., the US and Australia). The results in the literature have highlighted many topics related to knowing and using EBPs in those countries. However, the literature review revealed that no study has investigated teachers' knowledge and use of EBPs for students with ASD in developing countries. Thus, such a study would add to the literature by providing information on how teachers perceive their knowledge and use of EBPs in those countries. These insights will assist in identifying areas for further training and investigating generalization beyond developed countries. Therefore, this study was conducted to investigate the knowledge and use of EBPs to teach students with ASD by teachers in Saudi Arabia. This study attempted to answer four research questions: (i) to what extent do teachers know and use EBPs with students with ASD, (ii) to what extent do teachers differ in their reported knowledge and use of EBPs, (iii) what is the relationship between reported knowledge and use of EBPs, and (iv) what factors influence teachers' use of EBPs.

## Materials and Methods

### Participants

Participants were selected using probability sampling method with random sampling technique. They included 240 special education teachers in the city of Riyadh, Saudi Arabia. In this study, female and male teachers participated. Female teachers represented 53.3% of respondents and the remaining were male teachers. Most of the participants (72%) had majored in autism education, and the others were special education majors (24%) or general education majors with some professional development programs in special education (9%). The participants' experience with teaching students with autism ranged from <5 years to more than 10 years. Most of the participants, 94% and 95%, respectively, reported that they had <10 years of teaching experience or a bachelor's degree, and 5% reported that they had a master's degree. As for professional development programs, almost 9% of participants had not attended any training programs on autism, and the others had attended from two to more than five of some types of training programs. Table 1 presents the demographic information of the participants.

**Table 1:** Participants' characteristics.

**Variables**	**%**
**Gender**	
Male	47.7
Female	53.3
**Years of teaching experience with children with autism spectrum disorder**	
<5 years	50.8
Between 5 and 10 years	44.2
More than 10 years	5
**Highest degree earned**	
Bachelor	95
Master and above	5
**Professional development programs**	
None	9.2
2 programs or fewer	23.8
From 3 to 5 programs	33.3
More than 5 programs	33.8

### Instrument

The author developed the Teachers Knowledge and Use of EPBs Survey on the basis of a literature review (e.g., Paynter and Keen, 2015; Wong et al., 2015; Paynter et al., 2017). The survey comprised two parts. The first part of the survey collected demographic information: gender, major, highest degree earned (bachelor's degree, master's degree), overall years of teaching experience of students with ASD, and number of professional development programs attended. The second part of the survey contained a list of EBPs for teachers of students with ASD in educational settings and 26 items with forced-choice responses. The list was created by using the EPBs for children, youth, and young adults report developed by Wong et al. (2015). On the survey, each item included the name of the practice and its definition. The teachers read each item, identified their knowledge and use of it, and rated their knowledge and use of each practice on a 4-point scale from 0 = I don't know/never used to 3 = high knowledge/used frequently. The higher the score, the greater the knowledge and use of EBPs. The duration of the survey was ~10–15 min. Once the survey items were initially developed, several expert faculty members in the field of autism provided feedback on the clarity of the items and the constructs of the survey. They indicated that the survey was appropriate for the research purpose. In addition, the coefficient alphas for knowledge and use of EBPs were calculated separately. They demonstrated high reliability, with estimates of 0.93 for knowledge and 0.935 for use. These results indicated that the survey could be used as a valid, reliable tool for assessing the knowledge and use of EBPs for students with ASD.

### Procedures

The institutional review board (IRB) review was approved by the IRB committee in the school district, to ensure this study's compliance and ethical conduct of research involving human participants. After receiving IRB approval, public schools and private centers that provide services for ASD in Riyadh, Saudi Arabia, were identified. The two eligibility criteria for the participants were as follows: teachers in a public school or private center in Riyadh that provide services for students with ASD and who were teaching students with ASD at the time of the invitation to participate in the study. A coordinator at each site was contacted to introduce the purpose of the study, which included a brief description of the study, an informed consent form, demographic information, and the survey. The coordinators were further asked to distribute and collect the surveys. Participants were told that they were free to withdraw from the study at any point with no penalty. Two weeks later, follow-up reminders were sent to remind the coordinators to collect the surveys, which were then collected by the researcher.

### Data Analysis

Statistical Package of Social Science for Windows Version 22 was used to analyze the data. Preliminary analyses were conducted to determine whether the data was normally distributed using a variety of methods (Normal Q-Q Plots, histograms, and Shapiro-Wilk test). The results indicated that the data approximately normally distributed. In addition, there was homogeneity of variances, as assessed by Levene's test for equality of variances. Descriptive statistics (i.e., mean and standard deviations) were used to answer the first research question that measured teachers' knowledge and use of EBPs for students with ASD. Two independent sample *t*-tests and ANOVA were conducted to examine the differences in reported knowledge and use of EBPs on the basis of gender, teaching experience with students with ASD, and whether special education teachers had attended professional development programs. To measure the correlations between reported use of EBPs and teachers' knowledge of EBPs, Pearson's product-moment correlation coefficient was calculated. In addition, a stepwise multiple regression was used to test the predictability of several factors (gender, teaching experience of students with ASD, and professional development programs) for the teachers' use of EBPs.

## Results

### Teachers' Knowledge and Use of EBPs

Table 2 presents the teachers' mean scores of their perceived knowledge and self-reported use of each EBP and the overall means for knowledge and use of EBPs. The results indicated that the total mean for knowledge was 2.33, with item means ranging from 2.06 to 2.71. As for use, the total mean was 2.07, with item means ranging from 1.69 to 2.66. Generally, participants reported medium knowledge and use of EBPs for students with ASD. They agreed that the most commonly known and used EPBs were reinforcement (*M* = 2.71 for knowledge, *M* = 2.66 for use), prompting (*M* = 2.70 for knowledge, *M* = 2.62 for use), extinction (*M* = 2.61 for knowledge, *M* = 2.42 for use), and modeling (*M* = 2.55 for knowledge, *M* = 2.40 for use). However, there was variability in the least-known and least-used EPBs. Pivotal response training (*M* = 2.06), time delay (*M* = 2.07), scripting (*M* = 2.07), functional communication training (*M* = 2.12), and self-management (*M* = 2.17) were the least-known EPBs. The least-used EPBs were scripting (*M* = 2.69), social narratives (*M* = 2.70), self-management (*M* = 2.77), time delay (*M* = 2.82), and video modeling (*M* = 2.82).

**Table 2:** Mean Scores (M) and Standard Deviation (SD) of knowledge and use of evidence-based practices.

**Evidence-based practices**	**Knowledge**		**Use**	
	**M**	**SD**	**M**	**SD**
Antecedent-based intervention	2.25	0.745	1.99	0.816
Cognitive behavioral intervention	2.18	0.771	2.00	0.790
Differential reinforcement of alternative, incompatible, or other behavior	2.41	0.743	2.28	0.803
Discrete trial teaching	2.21	0.842	2.02	0.915
Exercise	2.35	0.740	2.05	0.844
Extinction	2.61	0.553	2.42	0.601
Functional behavior assessment	2.22	0.780	1.98	0.896
Functional communication training	2.12	0.840	1.87	0.960
Modeling	2.55	0.689	2.40	0.748
Naturalistic intervention	2.22	0.861	2.03	0.924
Peer-mediated instruction and intervention	2.38	0.744	1.95	0.932
Picture exchange communication system	2.41	0.743	2.09	0.935
Pivotal response training	2.06	0.904	1.87	1.004
Prompting	2.70	0.525	2.62	0.551
Reinforcement	2.71	0.508	2.66	0.540
Response interruption/redirection	2.43	0.705	2.25	0.780
Scripting	2.07	0.891	1.69	0.944
Self-management	2.17	0.823	1.77	0.986
Social narratives	2.20	0.836	1.70	0.956
Social skills training	2.33	0.763	2.08	0.902
Structured play group	2.24	0.822	1.88	0.933
Task analysis	2.45	0.701	2.27	0.832
Technology-aided instruction and intervention	2.37	0.748	2.10	0.860
Time delay	2.07	0.875	1.82	0.946
Video modeling	2.34	0.775	1.86	0.971
Visual support	2.48	0.702	2.24	0.853
Total	2.33	0.467	2.07	0.533

### Demographic Comparisons of Teachers

#### Gender

Saudi Arabia uses a single-education system: boys are educated by male teachers and girls are educated by female teachers. Thus, teachers' responses were compared based on their gender. An independent-samples *t-*test was conducted to evaluate that gender would be related to the teachers' reported knowledge and use of EBPs. Female teachers reported more knowledge of EBPs (*M* = 2.44, *SD* = 0.411) than male teachers did (*M* = 2.20, *SD* = 0.495), *t* (238) = −3.998, *p* = 0.000, *d* = 0.473. Female teachers also reported more use of EBPs (*M* = 2.24, SD = 0.453) than male teachers did (*M* = 1.88, SD = 0.554), *t*(238) = −5.549, *p* = 0.000, *d* = 0.652.

#### Teaching Experience

In this study, teachers were compared based on their experience with teaching students with ASD. They were placed into three groups (<5 years, from 5 to 10 years, more than 10 years). To determine whether the teaching experience would be related to the teachers' reported knowledge and use of EBPs, one-way ANOVA was conducted. Comparisons across teaching experience groups revealed no significant differences in reported knowledge of EBPs, [*F*_(2,237)_ = 2.31, *p* = 0.101, η*p*2 = 0.019]. The teachers were significantly different in reporting their use of EBPs, [*F*_(2,237)_ = 3.225, *p* = 0.042, η*p*2 = 0.026]. The Tukey honestly significant difference (HSD) test revealed that teachers with more than 10 years' experience in teaching reported more use of EBPs (*M* = 2.45, *SD* = 0.435) than did teachers with <5 years' experience (*M* = 2.06, *SD* = 0.496), *p* = 0.040, and teachers with between 5 and 10 years' experience (*M* = 2.05, *SD* = 0.571), *p* = 0.035. No significant differences were found between teachers with <5 years' experience and teachers with between 5 and 10 years' experience, *p* = 0.984.

#### Professional Development Programs

Teachers were divided into four groups: never attended professional development programs on teaching students with ASD, attended fewer than two programs, attended three to five programs, and attended more than five programs. To determine whether the professional development programs would be related to the teachers' reported knowledge and use of EBPs, one-way ANOVA was conducted. Comparisons across numbers of professional development programs attended revealed significant differences in reported knowledge of EBPs, [*F*_(3, 236)_ = 12.919, *p* = 0.000, η*p*2 = 0.141]. *Post-hoc* comparisons using Tukey HSD indicated that teachers who attended more than five programs reported knowing EBPs (*M* = 2.56, *SD* = 0.379) more than teachers who never attended professional development programs (*M* = 2.05, *SD* = 0.466), *p* = 0.000, teachers who attended fewer than two programs (*M* = 2.18, *SD* = 0.475), *p* = 0.000, and teachers who attended three to five programs (*M* = 2.28, *SD* = 0.450), *p* = 0.000. Other comparisons revealed no significant differences among teachers.

In addition, a significant difference was found in reported use of EBPs, [*F*_(3,236)_ = 16.785, *p* = 0.000, η*p*2 = 0.176]. *Post-hoc* comparisons using Tukey HSD indicated that teachers who attended more than five programs reported using EBPs (*M* = 2.37, SD = 0.467) more than teachers who never attended any programs (*M* = 1.80, SD = 0.492), p = 0.000, teachers who attended fewer than two programs (*M* = 1.87, SD = 0.513), p = 0.000, and teachers who attended three to five programs (*M* = 1.99, SD = 0.488), p = 0.000. Other comparisons revealed no significant differences among participants.

### Correlations Between Knowledge and Use of EBPs

Knowledge of EBPs was significantly correlated with the reported use of EBPs (*p* = 0.000). The strength of these correlations was large (*r* = 0.859). Thus, a high level of knowledge of EBPs could lead to high use of them.

### Factors Related to Teachers' Use of EBPs

Stepwise multiple regression was used to evaluate factors related to the use of EPBs. The predictors were gender, total years of experience teaching students with ASD, and total number of professional development programs on teaching students with ASD attended. In Table 3, the total number of professional development programs attended was entered into the regression equation in Step 1 of the analysis and was significantly related to the use of EBPs, [*F*_(1,238)_ = 42.143, *p* < 0.000]. In Step 2, the gender variable was added to the model and there was a significant change (*p* = 0.000), and *R*^2^ increased to 0.235. The total years of experience teaching students with ASD variable was excluded because it did not add to the predictability of the model. This result indicated that combination the of the total number of professional development programs attended and gender were the best predictors of EBPs' use. However, the use of EBPs was not affected by the total years of experience teaching students with ASD variables.

**Table 3:** Stepwise regression analysis of gender, total years of teaching experience, and total number of professional development programs attended on use of evidence-based practices (*N* = 240).

**Variable**	***B***	***SE B***	**β**
**Step 1**			
Constant	1.449	
Total number of professional development programs attended	0.214^**^	0.033	0.388
Adjusted *R^2^*	0.147
*F*	42.143^**^
**Step 2**			
Constant	1.028	
Total number of professional development programs attended	0.193^**^	0.032	0.351
Gender	0.314^**^	0.061	0.294
Adjusted *R^2^*	0.229
*F*	36.485^**^
*ΔR^2^*	0.085
F-change	26.340^**^

** *p < 0.01; B, unstandardized regression coefficient; SE, standard error; ß, standardized regression coefficient; ΔR^2^, difference in the proportion of variance explained*.

## Discussion

This study examined the levels of perceived knowledge and self-reported use of EBPs for students with ASD among special education teachers and the relationship between knowledge and use of EPBs. In addition, the group comparisons were conducted based on several variables (gender, total years of experience teaching students with ASD, and number of professional development programs attended). Factors related to teachers' use of EBPs (gender, total years of experience teaching children with ASD, and number of professional development programs attended) were also explored. The results of the study indicate that participants reported medium knowledge and use of EBPs for students with ASD. This finding revealed that participants have satisfactory levels of the theoretical and conceptual foundation of EPBs and the use of these practices. This finding might indicate that teachers receive information from pre- and in-service professional development programs on EPBs and that they translate this knowledge into practice at an adequate level. However, this is perceived knowledge and self-reported use. This means that teachers may think they know and use the practices but that they may not have accurate knowledge and actual use.

The findings from the teachers' responses to each EPB are consistent with those in the literature in that reinforcement (Paynter and Keen, 2015; Paynter et al., 2017) and modeling (Knight et al., 2019) were from the most known and used EPBs. However, the picture exchange communication system was not the most frequently used strategy, as indicated by the literature (Paynter and Keen, 2015; Paynter et al., 2017). The results confirm what has been found in the literature (Paynter and Keen, 2015; Paynter et al., 2017) in which self-management and time delay were the least-known and used EPBs. It appears that teachers know and use common practices. They also may prefer positive practices that are used easily and with a group of students. There may be more opportunity or need to use some practices (e.g., reinforcement) throughout the day. By contrast, teachers avoided some EBPs such as self-management, video modeling, scripting, and social narratives, which require time and energy to prepare and use inside the classroom. These practices might be more suitable for individual use than group interventions and be appropriately used less often in response to specific needs. Moreover, teachers reported less knowledge about functional communication training and pivotal response training (Paynter and Keen, 2015; Paynter et al., 2017; Knight et al., 2019) because these may not be popular and are rarely used in the classroom. The literature has suggested that teachers may not understand pivotal response training (Knight et al., 2019) and have difficulty using it (Stahmer et al., 2019).

When the teachers' responses were compared by gender, female teachers knew and used more of EBPs than their male counterparts did. Autism researchers have not compared teachers on the basis of this variable. However, this variable might be important in this research because females are educated and teach in separate schools. An explanation for this result could be that females have received more pre- and in-service professional development programs than males have; thus, females' knowledge has been increased and thus led to more use of these practices. Another explanation could be that female school principals encourage teachers to attend workshops and support using EBPs inside their schools. Males may depend in their teaching on guidance from experienced teachers or use common practices in their schools regardless of their effectiveness, and females prefer practices supported by scientific evidence. An alternative explanation might be that there are gender differences in self-perceptions or response styles rather than in actual knowledge or use of EBPs.

Participants were compared based on experience teaching students with ASD. The comparisons revealed that the teaching experience of students with ASD did not affect the reported knowledge of EBPs. By contrast, they affect reported use of EBPs, where teachers with more than 10 years' experience reported using more EBPs than did teachers with less experience. This finding could be logical in that experience may not increase a teacher's knowledge of EBPs because she may have received inadequate information on it. However, experience may help teachers use the practices because the more experienced teachers have attempted to implement many practices and may thus have gained confidence and skills in using practices, whereas teachers with less experience could have less confidence and fewer skills.

The number of professional development programs attended on teaching students with ASD influenced reported knowledge and use of EBPs. Teachers who attended more than five professional development programs were more knowledgeable and used more EBPs than teachers who attended fewer professional development programs. This finding emphasizes the importance of professional development programs in increasing teachers' knowledge and use of EBPs. When teachers attend more professional development programs, they know more about these practices and use them in their classes. Attending more programs will provide teachers opportunities to discuss their needs with other professionals and share their experiences in using these practices. This practice would facilitate the implementation of practices.

Knowledge of EBPs was significantly related to the reported use of EBPs. This finding suggests that when teachers have high levels of knowledge of EBPs, they use them more often. This finding is consistent with those in the literature (Cook et al., 2008; Paynter and Keen, 2015; Paynter et al., 2017; Hsiao and Sorensen Petersen, 2019). Research has suggested that teachers' knowledge influences the use of EBPs (Jones, 2009). This finding may suggest that teachers prefer to use familiar practices. By contrast, unfamiliar practices are rarely used regardless of the evidence supporting their use (Cook et al., 2008). In addition, knowledge might be a major barrier to the use of EBPs. For instance, some teachers may be confused between the meaning of best practice and EBPs. They may use practices that have not had strong evidence bases (Hornby et al., 2013). Ensuring teachers have sufficient knowledge related to teaching is vital for using that knowledge but does not guarantee that it will be used with fidelity (Scheeler et al., 2016).

As for the factors predicting teachers' use of EBPs, the combination of the number of professional development programs attended and gender were the best predictors, and the total years of experience teaching children with ASD variables did not predict the use of EBPs. This finding emphasizes the importance of in-service training programs because the results indicated that females attended more professional programs and had more knowledge and use of EBPs than males did. When teachers work with students, they may experience problems that entail attending training programs to find solutions. Subsequently, they attempt to use these solutions in their classroom.

The results of this study have implications. Because a strong positive relationship was observed between knowledge and use of EBPs, improving teachers' knowledge of EBPs for students with ASD could increase their use of that practice when teaching these students. Therefore, improving teachers' knowledge of EBPs for students with ASD is necessary. This practice might entail the inclusion of courses on EBPs in teacher education programs and how to use EBPs inside the classrooms. These courses should combine knowledge with opportunities to practice the knowledge and provide feedback and support on the implementation of EBPs. In addition, school principals and school district leaders should encourage and require teachers to attend professional development programs on EBPs. In this study, teachers needed to attend more than five professional development programs to influence their knowledge and use of EBPs. This finding may indicate the quality of the programs provided to teachers. Inferior programs may hinder the benefits. Notably, research revealed a scarcity of quality in-service training on EBPs for students with ASD (Alexander et al., 2015). When teachers attend high-quality programs, they might need to attend fewer programs to learn a lot about the practices and use them in their classrooms. This result may highlight the need for effective models of professional development programs. Research has indicated that most commonly used professional development approaches have little influence on teachers' knowledge and use of EBPs for students with ASD (Hornby et al., 2013; Brock et al., 2014). Evidence suggests that the most effective models of professional development programs, such as individualized coaching and mentoring, are not used widely in schools (Brock et al., 2014). Satisfactory professional development programs were consistently cited as successful facilitators of EBP use (Forman et al., 2009). Effective teacher training necessitates the inclusion of multiple approaches (Morrier et al., 2011). Using lectures and handouts alone to disseminate information on EBPs is often useless (Alexander et al., 2015). Didactic training could help spread information but may not lead to using that information (Morrier et al., 2011). Training teachers to use EBPs should advance beyond didactic instruction, to emphasize practicing EBPs inside the classroom and provide feedback on their implementation (Marder and deBettencourt, 2015). The programs should include hands-on activities and opportunities to practice and receive feedback from an expert coach or supervisor in the use of EBPs. Providing performance feedback is essential to effective implementation (Hall, 2015). Research has also indicated a need for follow-up training (Alexander et al., 2015) and ongoing support (Hornby et al., 2013; Hall, 2015) to increase the adoption of new practices and levels of implementation fidelity (Alexander et al., 2015) and to sustain the use of EBPs (Hornby et al., 2013; Hall, 2015). Training all teachers in a short time is difficult. Thus, school district leaders should develop handbooks and websites (Test et al., 2015) on EBPs with the appropriate information, for example, a description of each EBP, the steps and materials necessary for its implementation, and checklists to assess the fidelity of implementation. This effort will help reach teachers, regardless of location.

Although this study's results have revealed crucial findings on the knowledge and use of EBPs for students with ASD in Saudi Arabia, its limitations should be considered. First, the scope of this study was limited to a large city; thus, the results might not represent national findings. However, in the studied city, the teachers represent various backgrounds and cultures. Further research could conduct a national study that includes teachers from urban, suburban, and rural locations and subsequently compare their results with the results of this study. Further research could also investigate whether teachers working in different types of cities differ in their knowledge and use of EBPs or replicate this study and include participants from other fields such as occupational therapists and speech pathologists to explore if they differ in their knowledge and use of EPBs from the teachers in this study (Paynter et al., 2017).

Furthermore, this study used a survey to measure teachers' knowledge and use of EBPs for students with ASD. Thus, the results were self-reported, and teachers may have indicated that they had a higher level of knowledge and use EBPs than they did in practice; additionally, they may have inaccurate knowledge or use the practices with low fidelity. In addition, females reported more knowledge and use of EBPs than males did, and gender predicted teachers' use of EBPs. This finding highlights the need for further research that uses mixed-method research. Researchers could survey teachers on EPBs and observe teachers inside their classrooms to compare the self-reported results with actual use. They could also interview teachers to obtain in-depth information on their perceptions and use of EBPs.

Teachers' attitudes may play an important role in understanding EPBs. Personal views and attitudes may also strongly impact teachers' use of the practices (Jones, 2009; Paynter et al., 2017). Positive attitudes toward EBPs may facilitate high levels of knowledge and use of these practices, whereas negative attitudes may prevent teachers from using them. Encouraging positive attitudes toward EBPs may help enhance the knowledge and use of EBPs (Paynter and Keen, 2015). Thus, further research should study teachers' attitudes toward EBPs and how their attitudes influence their selection of practices and evaluate the effectiveness of training programs in positively changing teachers' attitudes toward EBPs and how this effort assists in enhancing teachers' knowledge and use of EBPs.

This study focused on a closed list of EBPs and thus excluded non-EBPs. The literature has indicated that participants frequently use non-EBPs (Hess et al., 2008; Burns and Ysseldyke, 2009; Carter et al., 2011, 2012). Thus, the teachers in this study may have used several non-EBPs, in addition to using EBPs. Thus, further research should measure and compare teachers' knowledge and use of EBPs and of non-EBPs, to provide a clear picture of the practices used inside the classroom. Sometimes, teachers need to know EPBs and non-EBPs so that they focus on using EBPs and avoid other practices. As a result, pre- and in-service training programs should provide information on EBPs and non-EBPs so that teachers understand the differences in the examples and non-examples of EBPs.

## Conclusions

This study is among the first to examine teachers' knowledge and use of EBPs in developing countries. The results indicate that teachers have a satisfactory level of knowledge and use of EBPs for students with ASD. Knowledge and use of EBPs were related. Gender and professional development programs were predictors of teachers' use of EBPs for students with ASD. This finding suggests that improving teachers' knowledge of EBPs for students with ASD could increase teachers' use of that practice. Thus, offering high-quality professional development programs could improve teachers' use of EBPs for students with ASD. Teachers should be encouraged and required to attend the professional development programs on EBPs. In addition, school district leaders should develop handbooks and websites (Test et al., 2015) on EBPs to reach teachers, regardless of location. However, this study assessed perceived knowledge and self-reported use; thus, further research could measure the actual knowledge and use of these practices.

## Data Availability Statement

The raw data supporting the conclusions of this article will be made available by the authors, without undue reservation.

## Ethics Statement

The study was conducted after approvals were granted from the College of Education at King Saud University and the General Administration of Education in Riyadh. Written informed consents were obtained from participants and were kept confidentiality. The patients/participants provided their written informed consent to participate in this study.

## Author Contributions

The author confirms being the sole contributor of this work and has approved it for publication.

## Funding

AA extend their appreciation to the Deanship of Scientific Research at King Saud University for funding this work.

## Conflict of Interest

The author declares that the research was conducted in the absence of any commercial or financial relationships that could be construed as a potential conflict of interest.

## Publisher's Note

All claims expressed in this article are solely those of the authors and do not necessarily represent those of their affiliated organizations, or those of the publisher, the editors and the reviewers. Any product that may be evaluated in this article, or claim that may be made by its manufacturer, is not guaranteed or endorsed by the publisher.

## References

[B1] Alexander J. L. Ayres K. M. Smith K. A. (2015). Training teachers in evidence-based practice for individuals with autism spectrum disorder: a review of the literature. Teach. Educ. Spec. Educ. 38, 13–27. 10.1177/0888406414544551

[B2] Alhossein A. (2016). Teachers' knowledge and use of evidence-based teaching practices for students with emotional and behavior disorders in Saudi Arabia. J. Educ. Pract. 7, 90–97. Available online at: https://files.eric.ed.gov/fulltext/EJ1126488.pdf

[B3] American Psychiatric Association (2013). Diagnostic and Statistical Manual of Mental Disorders (DSM-5^®^). Washington, DC: American Psychiatric Pub.

[B4] Beam H. D. Mueller T. G. (2017). What do educators know, do, and think about behavior? an analysis of special and general educators' knowledge of evidence-based behavioral interventions. Prevent. Sch. Failure 61, 1–13. 10.1080/1045988X.2016.1164118

[B5] Brock M. E. Huber H. B. Carter E. W. Juarez A. P. Warren Z. E. (2014). Statewide assessment of professional development needs related to educating students with autism spectrum disorder. Focus Autism Other Dev. Disabl. 29, 67–79. 10.1177/1088357614522290

[B6] Burns M. K. Ysseldyke J. E. (2009). Reported prevalence of evidence-based instructional practices in special education. J. Spec. Educ. 43, 3–11. 10.1177/0022466908315563

[B7] Carter M. Stephenson J. Strnadová I. (2011). Reported prevalence by Australian special educators of evidence-based instructional practices. Austr. J. Spec. Educ. 35, 47–60. 10.1375/ajse.35.1.47

[B8] Carter M. Strnadova I. Stephenson J. (2012). Reported prevalence of evidence-based instructional practices by special educators in the Czech Republic. Eur. J. Spec. Needs Educ. 27, 319–335. 10.1080/08856257.2012.691229

[B9] Cook B. G. Odom S. L. (2013). Evidence-based practices and implementation science in special education. Except. Child. 79, 135–144. 10.1177/001440291307900201

[B10] Cook B. G. Shepherd K. G. Cook S. C. Cook L. (2012). Facilitating the effective implementation of evidence-based practices through teacher-parent collaboration. Teach. Except. Child. 44, 22–30. 10.1177/004005991204400303

[B11] Cook B. G. Tankersley M. Harjusola-Webb S. (2008). Evidence-based special education and professional wisdom: putting it all together. Interv. Sch. Clin. 44, 105–111. 10.1177/1053451208321566

[B12] Forman S. G. Olin S. S. Hoagwood K. E. Crowe M. Saka N. (2009). Evidence-based interventions in schools: developers' views of implementation barriers and facilitators. Sch. Ment. Health 1:26. 10.1007/s12310-008-9002-5

[B13] Hall L. J. (2015). Sustaining evidence-based practices by graduated special educators of students with ASD: creating a community of practice. Teach. Educ. Spec. Educ. 38, 28–43. 10.1177/0888406414558883

[B14] Hess K. L. Morrier M. J. Heflin L. J. Ivey M. L. (2008). Autism treatment survey: services received by children with autism spectrum disorders in public school classrooms. J. Autism Dev. Disord. 38, 961–971. 10.1007/s10803-007-0470-5 17929155

[B15] Hornby G. Gable R. A. Evans W. (2013). Implementing evidence-based practice in education: what international literature reviews tell us and what they don't. Prevent. Sch. Failure Alternat. Educ. Child. Youth 57, 119–123. 10.1080/1045988X.2013.794326

[B16] Hsiao Y.-J. Sorensen Petersen S. (2019). Evidence-based practices provided in teacher education and in-service training programs for special education teachers of students with autism spectrum disorders. Teach. Educ. Spec. Educ. 42, 193–208. 10.1177/0888406418758464

[B17] Jones M. L. (2009). A study of novice special educators' views of evidence-based practices. Teach. Educ. Spec. Educ. 32, 101–120. 10.1177/0888406409333777

[B18] Kakkar D. (2019). Diagnostic assessment techniques and non-invasive biomarkers for autism spectrum disorder. Int. J. E-Health Med. Commun. 10, 79–95. 10.4018/IJEHMC.201907010530473705

[B19] Knight V. F. Huber H. B. Kuntz E. M. Carter E. W. Juarez A. P. (2019). Instructional practices, priorities, and preparedness for educating students with autism and intellectual disability. Focus Autism Other Dev. Disabl. 34, 3–14. 10.1177/1088357618755694

[B20] Lauderdale-Littin S. Brennan M. (2018). Evidence-based practices in the public school: the role of preservice teacher training. Int. Electron. J. Element. Educ. 10, 369–375. 10.26822/iejee.2018336195

[B21] Leblanc L. Richardson W. Burns K. A. (2009). Autism spectrum disorder and the inclusive classroom: effective training to enhance knowledge of ASD and evidence-based practices. Teach. Educ. Spec. Educ. 32, 166–179. 10.1177/0888406409334279

[B22] Maenner M. J. Shaw K. A. Baio J. (2020). Prevalence of autism spectrum disorder among children aged 8 years—autism and developmental disabilities monitoring network, 11 sites, United States, 2016. MMWR Surveill. Summ. 69:1. 10.15585/mmwr.ss6904a1 32214087 PMC7119644

[B23] Marder T. deBettencourt L. U. (2015). Teaching Students With ASD Using Evidence-Based Practices: Why Is Training Critical Now? Los Angeles, CA: SAGE Publications Sage.

[B24] Meindl J. N. Delgado D. Casey L. B. (2020). Increasing engagement in students with autism in inclusion classrooms. Child. Youth Serv. Rev. 111:104854. 10.1016/j.childyouth.2020.104854

[B25] Morrier M. J. Hess K. L. Heflin L. J. (2011). Teacher training for implementation of teaching strategies for students with autism spectrum disorders. Teach. Educ. Spec. Educ. 34, 119–132. 10.1177/0888406410376660

[B26] Odom S. L. Cox A. W. Brock M. E. A. S. D. N. P. D. C., on. (2013). Implementation science, professional development, and autism spectrum disorders. Except. Child. 79, 233–251. 10.1177/00144029130790020733732876

[B27] Paynter J. M. Ferguson S. Fordyce K. Joosten A. Paku S. Stephens M.. (2017). Utilisation of evidence-based practices by ASD early intervention service providers. Autism 21, 167–180. 10.1177/1362361316633032 27091949

[B28] Paynter J. M. Keen D. (2015). Knowledge and use of intervention practices by community-based early intervention service providers. J. Autism Dev. Disord. 45, 1614–1623. 10.1007/s10803-014-2316-2 25398604

[B29] Scheeler M. C. Budin S. Markelz A. (2016). The role of teacher preparation in promoting evidence-based practice in schools. Learn. Disabil. Contemp. J. 14, 171–187. Available online at: https://files.eric.ed.gov/fulltext/EJ1118433.pdf

[B30] Spooner F. Root J. R. Saunders A. F. Browder D. M. (2019). An updated evidence-based practice review on teaching mathematics to students with moderate and severe developmental disabilities. Remed. Spec. Educ. 40, 150–165. 10.1177/0741932517751055

[B31] Stahmer A. C. Suhrheinrich J. Roesch S. Zeedyk S. M. Wang T. Chan N.. (2019). Examining relationships between child skills and potential key components of an evidence-based practice in ASD. Res. Dev. Disabil. 90, 101–112. 10.1016/j.ridd.2019.04.003 31031082 PMC8109189

[B32] Stansberry-Brusnahan L. L. Collet-Klingenberg L. L. (2010). Evidence-based practices for young children with autism spectrum disorders: guidelines and recommendations from the national resource council and national professional development center on autism spectrum disorders. Int. J. Early Child. Spec. Educ. 2, 45–56. 10.20489/intjecse.107957

[B33] Test D. W. Kemp-Inman A. Diegelmann K. Hitt S. B. Bethune L. (2015). Are online sources for identifying evidence-based practices trustworthy? an evaluation. Except. Child. 82, 58–80. 10.1177/0014402915585477

[B34] Wadhera T. Kakkar D. (2020). Interdisciplinary Approaches to Altering Neurodevelopmental Disorders. (AMDTC Book Series). Hershey. PA: IGI Global.

[B35] Wadhera T. Kakkar D. Rani R. (2021). Behavioral modeling using deep neural network framework for ASD diagnosis and prognosis, in Emerging Technologies for Healthcare: Internet of Things and Deep Learning Models, eds Mangla M. Sharma N. Mittal P. Wadhwa V. M. Thirunavukkarasu K. Khan S. (Hoboken, NJ: Wiley), 279–298.

[B36] Wong C. Odom S. L. Hume K. A. Cox A. W. Fettig A. Kucharczyk S.. (2015). Evidence-based practices for children, youth, and young adults with autism spectrum disorder: a comprehensive review. J. Autism Dev. Disord. 45, 1951–1966. 10.1007/s10803-014-2351-z 25578338

